# Adoptive Cell Therapy in Mice Sensitized to a Grass Pollen Allergen

**DOI:** 10.3390/antib13020048

**Published:** 2024-06-18

**Authors:** Anna Marianne Weijler, Lisa Prickler, Verena Kainz, Eva Bergmann, Barbara Bohle, Heinz Regele, Rudolf Valenta, Birgit Linhart, Thomas Wekerle

**Affiliations:** 1 Division of Transplantation, Department of General Surgery, Medical University of Vienna, 1090 Vienna, Austria; anna.weijler@meduniwien.ac.at (A.M.W.); lisa.prickler@meduniwien.ac.at (L.P.); verena.kainz@meduniwien.ac.at (V.K.); evabergmann08@web.de (E.B.); 2 Department of Pathophysiology and Allergy Research, Center for Pathophysiology, Infectiology and Immunology, Medical University of Vienna, 1090 Vienna, Austria; barbara.bohle@meduniwien.ac.at (B.B.); rudolf.valenta@meduniwien.ac.at (R.V.); 3 Clinical Institute of Pathology, Medical University of Vienna, 1090 Vienna, Austria; heinz.regele@meduniwien.ac.at; 4 Karl Landsteiner University of Health Sciences, 3500 Krems, Austria; 5 Institute of Immunology Federal Medical-Biological Agency (FMBA) of Russia, National Research Center (NRC), 119435 Moscow, Russia; 6 Laboratory of Immunopathology, Department of Clinical Immunology and Allergy, Sechenov First Moscow State Medical University, 119991 Moscow, Russia

**Keywords:** cell therapy, allergy, Phl p 5, tolerance, mouse model

## Abstract

The proportion of patients with type I allergy in the world population has been increasing and with it the number of people suffering from allergic symptoms. Recently we showed that prophylactic cell therapy employing allergen-expressing bone marrow (BM) cells or splenic B cells induced allergen-specific tolerance in naïve mice. Here we investigated if cell therapy can modulate an established secondary allergen-specific immune response in pre-immunized mice. We sensitized mice against the grass pollen allergen Phl p 5 and an unrelated control allergen, Bet v 1, from birch pollen before the transfer of Phl p 5-expressing BM cells. Mice were conditioned with several combinations of low-dose irradiation, costimulation blockade, rapamycin and T cell-depleting anti-thymocyte globulin (ATG). Levels of allergen-specific IgE and IgG1 in serum after cell transfer were measured via ELISA and alterations in cellular responses were measured via an in vitro proliferation assay and transplantation of Phl p 5^+^ skin grafts. None of the tested treatment protocols impacted Phl p 5-specific antibody levels. Transient low-level chimerism of Phl p 5^+^ leukocytes as well as a markedly prolonged skin graft survival were observed in mice conditioned with high numbers of Phl p 5^+^ BMC or no sensitization events between the day of cell therapy and skin grafting. The data presented herein demonstrate that a pre-existing secondary allergen-specific immune response poses a substantial hurdle opposing tolerization through cell therapy and underscore the importance of prophylactic approaches for the prevention of IgE-mediated allergy.

## 1. Introduction

Over one third of the world’s population are affected by immunoglobulin E (IgE)-mediated allergy, a number, that is gradually increasing in industrialized countries [1,2]. Individuals experiencing IgE-mediated allergy tend to react to environmental allergens, influenced by a number of hereditary and environmental factors. Even though no single chromosomal region or gene has been found to be linked with all phenotypes of type I allergy, polymorphisms in specific genes have been identified to affect individual susceptibility to allergies [3,4,5,6]. IgE sensitization occurs early in life, and recent birth cohort studies suggest that it may be initiated by a relatively small number of allergens depending on the living environment [1,7]. Continuous allergen contact shifts preclinical IgE sensitizations to the onset of moderate symptoms, such as rhinoconjunctivitis, which may progress to severe and chronic allergic reactions such as allergic asthma [8,9,10,11,12]. The natural course of allergies would strongly argue in favour of prophylactic treatments. Indeed, strategies like oral tolerance induction and vaccination are under investigation for allergy prevention within a time window after birth, which has yet to be determined [13,14,15]. However, up to now no allergy prophylaxis is available. 

For therapy of already sensitized patients suffering from allergic manifestations, several causative treatments such as allergen-specific immunotherapy or biologics which counteract allergic inflammation, are in use. While the latter are restricted to patients with severe symptoms and connected with high costs, allergen-specific immunotherapy has shown long-lasting beneficial effects. However, current allergy vaccines can induce severe side effects and clinical improvement of treated patients may vary [14,16]. 

We have developed a robust and long lasting protocol for prophylactic induction of antigen-specific tolerance, which is based on the transplantation of donor hematopoietic stem cells (HSC). This approach has successfully led to tolerance-induction in the setting of organ transplantation in various pre-clinical models, including mice and non-human primates (NHP), as well as in selected pilot clinical trials involving kidney transplantation [17,18]. Implementing this approach in allergy research, our group has previously demonstrated, that the transfer of allergen-expressing bone marrow (BM) cells or splenic lymphocytes can induce allergen-specific tolerance and long-lasting (BM cell transfer) or transient (B cell transfer) chimerism preventing the production of allergen specific IgE as well as IgG1 antibodies [19,20]. It is currently not known if this approach can be used for the treatment of an already established allergic immune response. Results from the fields of allotransplantation and autoimmunity indicate that pre-existing B and T cell responses in already sensitized recipients represent a major hurdle impeding antigen-specific tolerance induction [21,22]. Consequently, different conditioning protocols have been described in murine transplantation models to overcome antigen-specific sensitization and allow graft survival [23,24]. Of note, clinical studies performing autologous HSC transplantation in patients suffering from the autoimmune disease multiple sclerosis demonstrated a decrease in disease activity as well as an increase in patients’ quality of life, indicating a promising possibility to overcome pre-existing antigen-specific immune responses, which however requires relatively harsh conditioning [25,26]. 

Here we investigated, if tolerization of secondary allergen-specific immune responses is possible in murine recipients pre-immunized to a clinically relevant allergen by using allergen-specific cell therapy [19,20]. In contrast to tolerance induction or de-sensitization studies carried out in transplantation research involving multiple targets, such as minor and major histocompatibility antigens, our model focusses on a single antigen. 

To this end, we sensitized mice against the major grass pollen allergen, Phl p 5. Treatment with Phl p 5-expressing BM cells was combined with different conditioning regimens, including costimulation blockade, rapamycin, irradiation, and anti thymocyte globulin (ATG). We found that transient low-level chimerism can be induced in mice receiving ATG treatment. Ablation of humoral responses, however, could not be achieved. Skin graft survival was prolonged in recipients of high doses of allergen-expressing BM cells, or in pre-immunized individuals which did not receive allergenic stimulation after the treatment regimen.

## 2. Materials and Methods

### 2.1. Mouse Strains

Female BALB/c mice were purchased from Janvier-Labs (France) (6–12 weeks old). Numbers of mice used for every group are listed in Table 1. Phl p 5-transgenic mice (BALB/c background) were bred at the animal facility of the Medical University of Vienna. This mouse strain ubiquitously expresses the major timothy grass pollen allergen Phl p 5 on the cell surface and co-expresses eGFP intracellularly, which has been described previously [27]. Mice were housed under barrier conditions at the Medical University of Vienna’s animal facility. The Austrian Federal Ministry of Education, Science, and Research’s ethical votum granted approval for all experiments (permission numbers: 2021-0.276.441, approved on the 24 September 2021; BMWFW-66.009/0118-WF/V/3b/2016, approved on the 22 April 2016; BMWFW-66.009/0028-WF/V/3b/2015, approved on the 2 February 2015), and all studies were conducted in compliance with both international and national standards for the care of laboratory animals.

### 2.2. Recombinant Allergens

Recombinant Phl p 5 and Bet v 1 were produced and purified as previously described [28,29].

### 2.3. Immunization of Mice

Immunization was performed by subcutaneous (s.c.) injection of 5 × 10^6^ splenocytes isolated from Phl p 5^+^ mice and 5 µg recombinant Bet v 1 as control allergen, similar as described in [19]. Phl p 5^+^ splenocytes as well as Bet v 1 were adsorbed to aluminium hydroxide according to the manufacturer’s instructions (Alu-Gel-S; Serva, Ingelheim, Germany). Blood samples were collected repeatedly for serum isolation before and after immunization events.

### 2.4. Determination of Allergen-Specific Antibody Responses by ELISA

Allergen-specific IgE and IgG_1_ antibodies were measured via ELISA. Therefore, maximum adsorbance plates (Thermo Fisher Scientific, Waltham, MA, USA) were coated with 5 µg/mL Phl p 5 or Bet v 1. For IgE detection, serum was diluted 1/20, whereas for IgG_1_ detection, serum was diluted 1/500. Allergen-bound antibodies were detected using a monoclonal rat anti-mouse IgG_1_ (clone A85-1) and IgE (clone R35–72) antibodies (BD, San Diego, CA, USA) diluted 1/1000, followed by incubation with a horseradish peroxidase (HRP)-coupled goat anti-rat antiserum (Biosciences, Amersham, UK) diluted 1/2000. ABTS (2,2′-azino-bis(3-ethylbenzothiazoline-6-sulfonic acid) was used as substrate for HRP. Absorption (optical density) was measured using a Tecan Infinite F50 plate reader at an extinction of 405 nm, whereas background measurement was done at 492 nm.

### 2.5. Bone Marrow Transplantation Protocol

Hip bone, femur, tibia and humerus of Phl p 5^+^ donor mice were isolated and BM cells were flushed using PBS-/-. 25 × 10^6^ BMC in Medium 199 supplemented with HEPES and Gentamicin were injected per mouse intravenously (i.v.) into the tail vein on day 0 and, where indicated, mice received a second dose of 25 × 10^6^ BMC on day 5. Where indicated, the T cell depletion agent anti-thymocyte globulin (ATG) was injected intraperitoneally (i.p.) (0.45 mg/mouse) on day −2. Two Gy irradiation was given on d −1 and 0.1 mg rapamycin (LC Laboratories, Woburn, MA, USA) on day −1, day 0 and on day 2 after BM transplantation (for experimental groups see Table 1). Additionally, mice received costimulation blockade consisting of anti-CD40L (MR1; 1 mg, day 0; BioXcell, Lebanon, NH, USA) and CTLA4Ig (0.5 mg, day 2; abatacept, Bristol-Myers Squibb Pharmaceuticals, Princeton, NJ, USA).

### 2.6. Flow Cytometry Analysis of Chimerism

Heparinized blood was collected and red blood cells were removed through osmotic lysis via H_2_O_dd_. Lysis was stopped by adding 10x HANKS solution. Cells were stained for flow cytometric analysis using the following antibodies: CD19-APC-Cy7 (clone: 6D5), CD11b-PE (clone: M1/70), CD4-PE-Cy7 (clone: GK1.5), CD8-APC (clone: 53-6-7), CD3-eFluor450 (clone: 17A2) and CD45.2-eFluor500 (clone: 104). Exclusion of dead cells was done using 7AAD (Viability Staining Solution, BioLegend, San Diego, CA, USA). The frequency of Phl p 5^+^ cells was determined by measuring the co-expression of GFP in transferred donor cells, as shown previously [19]. Flow cytometric analyses were performed using a BD FACS Canto II (BD Biosciences, San Diego, CA, USA), and data were analysed using FlowJo software version 10.7.2.

### 2.7. Rat Basophil Leukemia Cell Degranulation Assay

IgE-specific mediator release of a rat basophil leukemia (RBL) cell line was measured as described previously [27]. Briefly, RBL-2H3 cells were transferred into 96-well tissue culture plates at a concentration of 4 × 10^4^ cells/well and incubated for 24 h at 37 °C, 5% CO_2_. Subsequently cells were loaded with mouse sera (diluted 1:40 with complete medium) for 2 h at 37 °C, 5% CO_2_. After incubation, cells were washed with Tyrode’s buffer followed by incubation with 0.01 µg/well rPhl p 5 for 30 min at 37 °C. Supernatants were transferred to fresh plates and incubated with 80 µM 4-methylumbelliferyl-N-acetyl-b-D-glucosamide (Merck, Darmstadt, Germany) in citrate buffer for 1 h at 37 °C. The reaction was stopped via addition of Glycine buffer followed by measurement of fluorescence at λex:360/λem:465 nm using a fluorescence microplate reader (Tecan Spark, Tecan Trading AG, Männedorf, Switzerland). Percentage of release was calculated by using degranulation after the addition of 10% Triton X-100 (Merck, Darmstadt, Germany) as total (100%) β-hexosaminidase release.

### 2.8. Skin Grafting

The skin (cutis and subcutis) was dissected from the tail of Phl p 5^+^ transgenic mice, cut into sufficiently large square pieces and stored in cool, sterile PBS-/-. Recipients were anaesthetized using Ketamin (100 mg/kg), Xylazin (5 mg/kg) und Buprenorphin (0.12 mg/kg). The left flank of the anaesthetized recipient was shaved and a piece of skin slightly larger (10%) than the skin graft was removed using scissors. The rectangular grafts were sutured to the dorsal thoracic wall at each corner and fixed with plasters.

### 2.9. Histological Analysis

Sections were cut from paraffin-embedded tissue fixed in 4.5% formalin (buffered pH of 7.5), stained with hematoxilin and eosin (HE) according to standard protocol, and analysed by an experienced pathologist blinded to the treatment conditions. Scans of pathology slides were taken using a TissueFAXS (Zeiss, Jena, Germany) at 20× magnification.

### 2.10. Mixed Lymphocyte Reaction (MLR)

Responder cells isolated from lymph nodes of experimental or naïve BALB/c mice were stained with violet proliferation dye for tracing proliferation. 4 × 10^5^ VPD^+^ responder cells were incubated with 4 × 10^5^ irradiated (30Gy) stimulatory cells (splenocytes) isolated from Phl p 5^+^ mice. After 5 days of culture, Phl p 5-specific proliferation was analysed for CD4^+^ and CD8^+^ cells using flow cytometry (BD FACS CANTO II).

### 2.11. Statisical Analysis

The statistical analyses were performed using GraphPad Prism 5.0 (GraphPad Software, Inc., San Diego, CA, USA). Box-Plots are presented as Tukey Plots representing 50% of the values within the boxes and indicated median values. Whiskers of the Box Plots represents 1.5-fold of the interquartile range (IQR). Data outside of the IQR are represented as dots. Dot plots indicate mean values with standard deviation (SD). *p*-values below 0.05 were considered statistically significant. The depicted *p*-values were calculated using a two-sided Mann-Whitney U test. Skin graft survival between two groups was compared using a log-rank test.

## 3. Results

### 3.1. Injection of Phl p 5^+^ BM Cells in Pre-Immunized Mice Significantly Boosted the Phl p 5-Specific IgE but Not IgG_1_ Response despite Immune Suppression

In previous studies we demonstrated that the transfer of allergen-expressing immune cells to naïve recipient mice induces robust allergen-specific tolerance, fully preventing the onset of allergen-specific immune responses. This current work investigates the potential of our cell therapy approach to modulate pre-existing allergic sensitization in a therapeutic model.

In a first attempt we investigated the effect of cell therapy in combination with different conditioning protocols on allergen-specific antibody responses. Naive BALB/c mice were sensitized s.c. to Phl p 5 as well as to a control allergen, Bet v 1, 15 and 12 weeks before the transfer of Phl p 5^+^ bone marrow cells. All mice developed Phl p 5- and Bet v 1-specific IgG_1_ antibodies (Supplementary Figure S1). 

**Figure 1:**
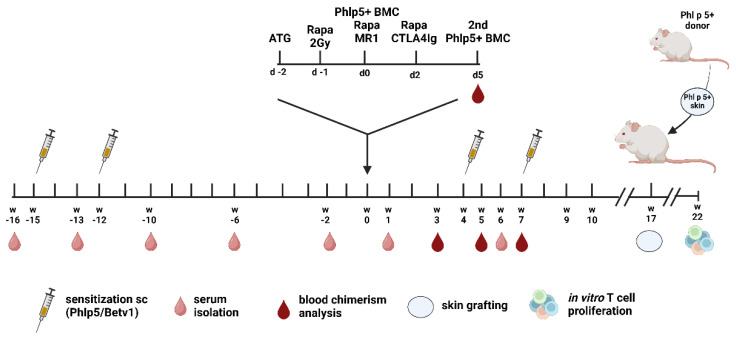
**Experimental Timeline** BALB/c mice were sensitized to Phl p 5 and Bet v 1 in weeks −15 and −12 before and 4 and 7 weeks after cell therapy. On day 0, 25 *×* 10^6^ Phl p 5^+^ BM cells were injected i.v. One experimental group received a second dose of 25 × 10^6^ BM cells on day 5. Mice were pre-treated with a single dose of ATG (0.45 mg, day −2) and 2Gy irradiation on day −1. Additionally, they received costimulation blockade (1 mg MR1, 0.5 mg CTLA4Ig) and a short course of Rapamycin (0.1 mg, day −1, d0, d2). 17 weeks after treatment, mice received a Phl p 5^+^ skin allograft and 22 weeks later mice were sacrificed for in vitro analysis of Phl p 5-specific T cell proliferation. Remaining skin grafts were histologically analysed. (ATG, anti-thymocyte globulin; BMC, bone marrow cells; sc, subcutaneous).

Thereupon, all experimental groups were injected i.v. with 25 × 10^6^ Phl p 5^+^ BM cells and were treated with a short course of rapamycin, costimulation blockade consisting of anti-CD154 and CTLA4Ig, as well as non-lethal irradiation (2Gy). In order to determine, if the amount or increased frequency of injected BM cells is of importance in this model, similar to a tolerance model depicted by Larsen et al., selected mice were injected with 50 × 10^6^ Phl p 5^+^ BM cells divided into two doses on day 0 and day 5 (Group C) [30]. Furthermore, to explore the necessity of T cell depletion, all but one group received ATG two days before the Phl p 5^+^ BM cell transfer (Group B). All experimental groups except for one received two additional immunizations with the allergens after the therapy regimens. The experimental timeline is depicted in Figure 1 and the detailed treatment regimen of the different experimental groups are depicted in Table 1.

**Figure 2:**
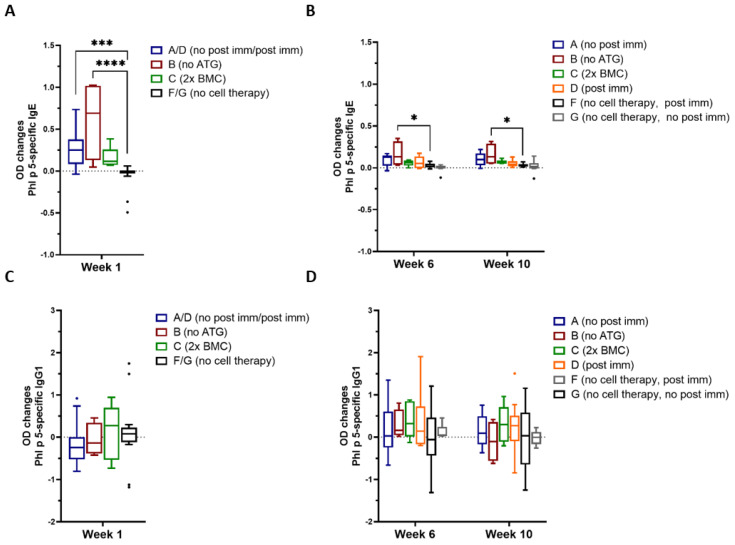
**Changes in Phl p 5-specific IgG1 and IgE levels after cell therapy** Phl p 5-specific IgE (**A**,**B**) and IgG_1_ (**C**,**D**) levels (*y*-axes) were measured via ELISA at different time points (*x*-axes). Changes of antibody levels compared to levels before treatment are shown. Therefore, OD values of antibody levels in serum isolated 1, 6 and 10 weeks after cell transfer were subtracted with OD values measured in serum 2 weeks before cell transfer. As group A and D, as well as F and G received the same treatment up to the depicted time point week 1 after cell transfer, values of these groups were pooled. A n = 9, B n = 4, C n = 5, D n = 10, F n = 6, G n = 10; Results are presented as Tukey-Box-Plots with indicated median, significant differences between groups are indicated and *p* values are shown. (* *p* < 0.05, *** *p* < 0.001, **** *p* < 0.0001) (ATG, anti-thymocyte globulin; BMC, bone marrow cells; immunization, immune).

Phl p 5- as well as Bet v 1-specific antibodies in murine sera were measured via ELISA. As shown in Figure 2A, significantly increased levels of Phl p 5-specific IgE, but not IgG1 (Figure 2C), were detectable within one week after cell therapy compared to controls without cell transfer (Group F and G). Interestingly, mice having received cell therapy without ATG (Group B) showed the highest Phl p 5-specific IgE levels until the end of the observation time frame of week 10 after cell transfer, which was again not observed for Phl p 5-specific IgG_1_ (Figure 2B,D). Furthermore, Bet v 1-specific IgE remained unaltered while Bet v 1–specific IgG1 levels even declined (Supplementary Figure S2).

**Figure 3:**
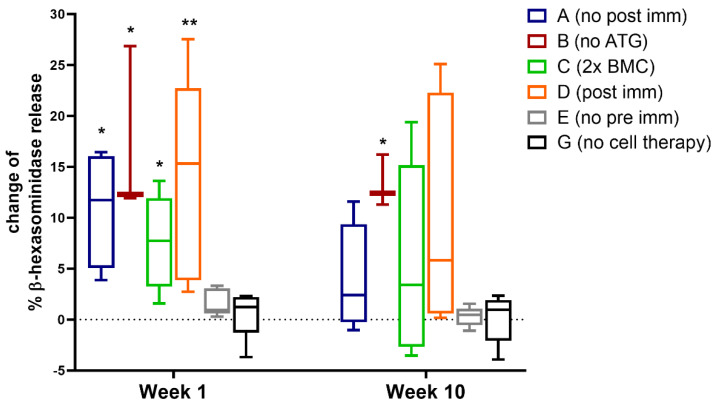
**Functional analysis of Phl p 5-specific IgE via a RBL degranulation assay** RBL cells were loaded with sera from different treatment groups collected at the indicated time points (*x*-axis) and challenged with rPhl p 5. Changes of the percentages of total ß-hexosaminidase release compared to values of week −2 are shown (*y*-axis). A n = 4, B n = 3, C n = 5, D n = 5, E n = 5, G n = 5; statistics was calculated on values of of Group G (no cell therapy), Results are presented as Tukey-Box-Plots with indicated median, significant *p* values are shown. (* *p* < 0.05, ** *p* < 0.01) (ATG, anti-thymocyte globulin; BMC, bone marrow cells; immun, immunization).

As a next step, we investigated, if our protocol had an effect on the functional activity of Phl p 5-specific IgE. Deploying a RBL degranulation assay we measured activation and subsequent effector release of IgE effector cells in vitro. Similar to the observation of Phl p 5-specific IgE levels after cell transfer, we observed significantly increased mediator release at week 1 in groups receiving the Phl p 5-specific cell therapy, despite strong immune suppression (Figure 3). Furthermore, RBL cells loaded with sera from pre-immunized mice which did not receive cell therapy (Group G) did not show increased degranulation in week 1, suggesting, that the intravenous injection of Phl p 5^+^ BM cells is responsible for the increased effector cell activation in the experimental groups. In accordance with previous studies, demonstrating that the transfer of Phl p 5-expressing BMC into naive recipients prevented Phl p 5-specific sensitization, no Phl p 5-induced degranulation from RBL cells was detected in group E (Figure 3). 

### 3.2. Transient Low-Level Chimerism Is Induced in Pre-Immunized Mice and Is Dependent on ATG Treatment

We further investigated the influence of different conditioning regimens on the survival of the injected Phl p 5^+^ BM cells by measuring Phl p 5-specific chimerism. 

**Figure 4:**
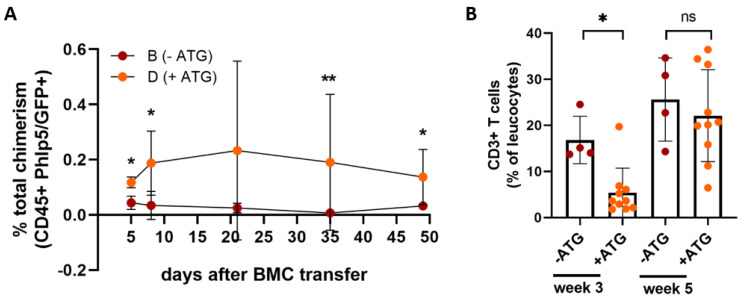
**Addition of ATG for donor BM survival** (**A**) mice treated with a single dose of ATG developed a transient mixed chimerism with Phl p 5^+^ leukocytes. (**B**) T cell depletion of mice with ATG in their treatment regimen compared to mice receiving cell therapy without ATG. B n = 4, D n = 10; Results are presented as mean values + SD, significant *p* values are shown. (* *p* < 0.05, ** *p* < 0.01; ns, non-significant; ATG, anti-thymocyte globulin; SD, standard deviation).

First we compared chimerism levels in mice having received cell therapy with or without T cell depletion by means of ATG treatment (Groups B, D) (Figure 4A). In the ATG-treated group, Phl p 5^+^ cells were detectable in the peripheral blood for at least 7 weeks after cell transfer compared to the mice which did not receive ATG, with no detectable Phl p 5^+^ cells present. Figure 4B shows the significant decrease of CD3^+^ T cell levels in ATG treated mice compared to mice whose treatment protocol did not include ATG.

**Figure 5:**
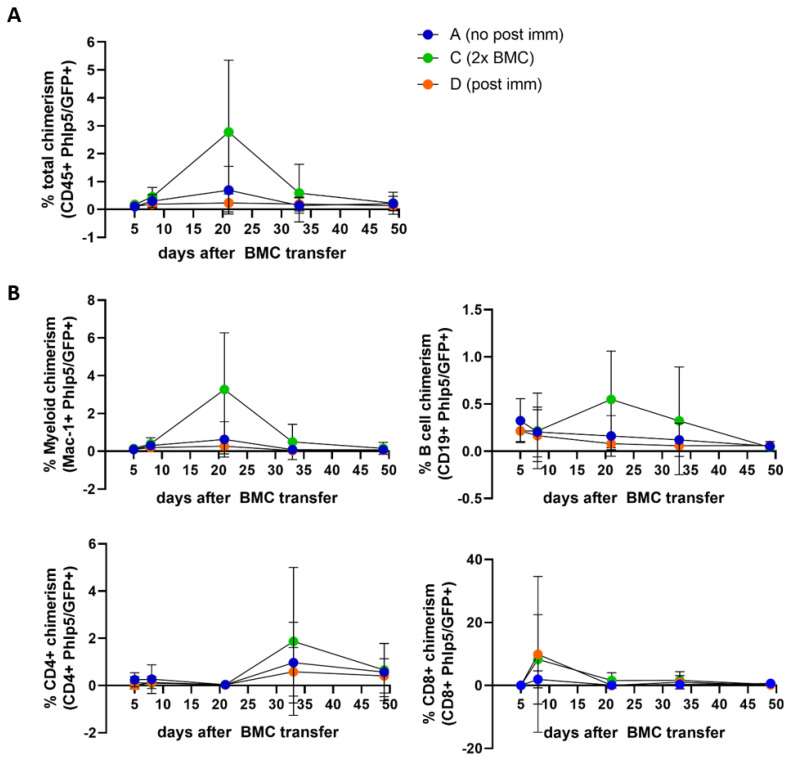
**Development of transient Phl p 5/GFP^+^ donor chimerism** (**A**) Percentage of Phl p 5/GFP^+^ cells in CD45^+^ cell population in different experimental groups. (**B**) Lineage-specific Phl p 5/GFP-specific chimerism of myleoid CD11b^+^ cells, CD19^+^ B cells, CD4^+^ and CD8^+^ T cells. A n = 9, C = 5, D = 10; Results are presented as mean values + SD (BMC, bone marrow cells; immun, immunization; SD, standard deviation).

To compare chimerism levels in the experimental groups which received ATG two days before cell transfer (Group A, C and D), we depicted percentages of Phl p 5^+^ cells over time in the peripheral blood in Figure 5. Phl p 5^+^ CD45^+^ cells were detectable at early time points, but decreased by week 5 (Figure 5A). A strong trend towards higher chimerism levels was observed in mice receiving a higher BM cell dose (2 × 25 × 10^6^ versus 1 × 25 × 10^6^). We further analyzed different subsets of Phl p 5^+^ cells in the recipient mice (myeloid cells, B cells, CD4^+^ and CD8^+^ T cells). Mainly myeloid (CD11b^+^) and B cells (CD19^+^) from donor mice were transiently detectable (Figure 5B). Interestingly, we saw a short, but profound increase in the percentage of Phl p 5^+^ CD8^+^ T cells in individual mice on day 8. This can be explained by the almost non-existing recipient CD8^+^ T cells following ATG depletion. As a side note, the naïve control mice receiving Phl p 5^+^ cells in combination with costimulation blockade, rapamycin as well as ATG and low-dose irradiation (Group E), developed robust, long-lived chimerism levels, indicating that the transient state of chimerism in our experimental mice is due to the Phl p 5-specific pre-immunization and not due to the treatment protocol itself (Supplementary Figure S3).

### 3.3. Influence of Phl p 5^+^ BM Cell Therapy on In Vitro T Cell Proliferation and Survival of Phl p 5^+^ Skin Grafts

**Figure 6:**
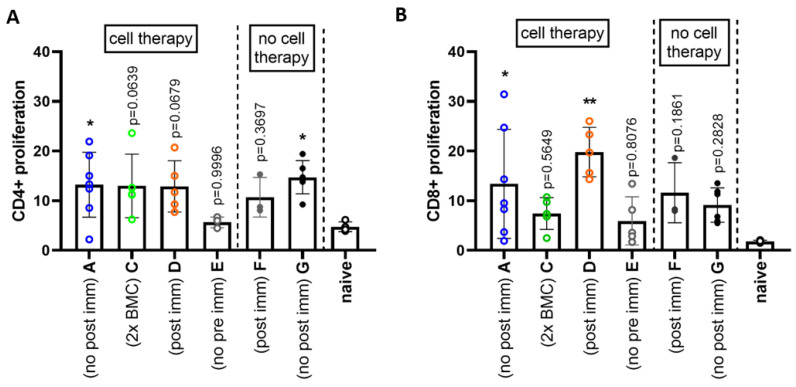
**Measurement of Phl p 5-specific T cell proliferation** Mixed lymphocyte reaction results from all experimental groups after Phl p 5^+^ BMC transfer. The graph in (**A**) depicts proliferation of CD4+ T cells while (**B**) shows proliferation of CD8+ T cells in the same sample. A n = 7, C n = 5, D n = 5, E n = 5, F n = 3, G n = 6, Results are presented as mean values + SD, statistics was calculated on values of naïve mice, all *p* values are shown (* *p* < 0.05, ** *p* < 0.01; BMC, bone marrow cells; immun, immunization; SD, standard deviation).

Besides alterations in the development of Phl p 5-specific antibodies, we investigated possible changes in T cell reactivity against Phl p 5. Therefore, we carried out an in vitro proliferation assay. Lymph node cells from all mouse groups were isolated in week 22 after Phl p 5^+^ BM cell transfer and co-cultured with irradiated splenocytes from Phl p 5^+^ donor mice. As shown in Figure 6A CD4^+^ T cell proliferation, determined by VPD staining, was elevated in all presensitized groups compared to naive mice, suggesting a recall response of initially primed T cells. However, only groups A (presensitized + cell therapy) and G (only presensitized) reached significance. Interestingly, CD8^+^ T cell proliferation was lower in mice receiving two doses of Phl p 5^+^ BM cells and therefore a higher number of Phl p 5^+^ BM cells (Group C) (Figure 6B).

**Figure 7:**
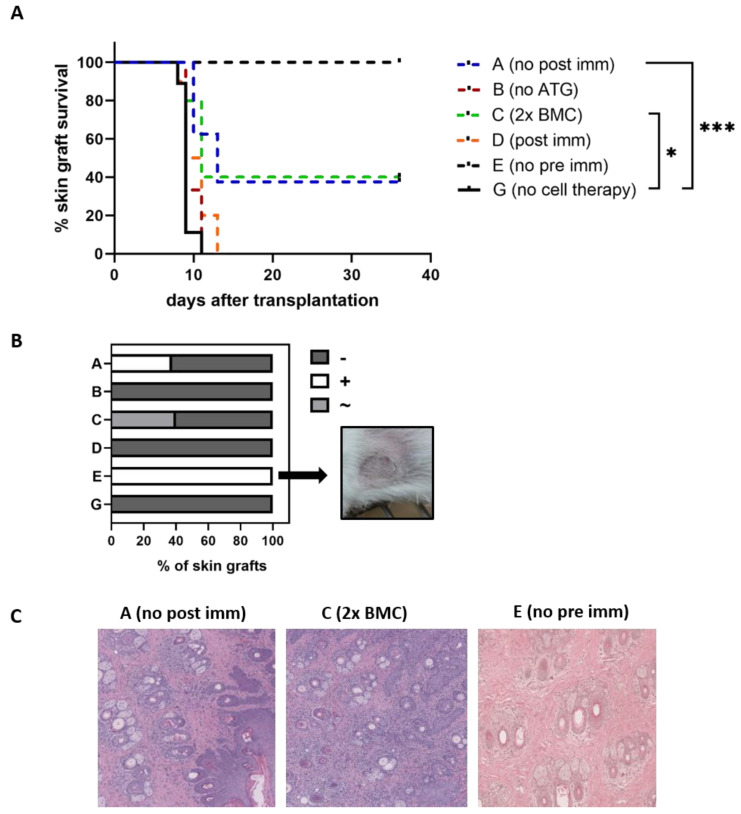
**Skin graft survival in pre-immunized mice for detection of allergen-specific cellular reactivity** (**A**) Mice of different experimental groups received a Phl p 5^+^ skin graft between 11 and 17 weeks after the BMC transfer regimen. Survival of skin grafts is depicted as Kaplan-Meier curve and survival probability is compared via a log-rank test. (**B**) Visual evaluation of remaining skin grafts at point of take down 5 weeks after skin grafting with + representing vital skin grafts, without lesions and hair growth, ~representing vital skin graft and lesions, and-representing rejected skin grafts. (**C**) HE staining of remaining skin grafts at take down (20× magnification). (* *p* < 0.05, *** *p* < 0.001; ATG, anti-thymocyte globulin; BMC, bone marrow cells; HE, hematoxilin and eosin; immune, immunization).

Additionally, we transplanted Phl p 5^+^ skin grafts on our experimental as well as control mice in order to investigate further (T cell-mediated) Phl p 5-specific immune responses in vivo. As shown in Figure 7A, skin graft survival was prolonged in mice treated with an increased dose of Phl p 5^+^ BM cells (Group C) and in mice which were not sensitized with Phl p 5 after the BM cell transfer (Group A). Surviving skin grafts were isolated 5 weeks after skin grafting and evaluated visually (Figure 7B) as well as via H&E staining by an experienced pathologist (Figure 7C). Control mice which developed a robust chimerism (Group E), did not show any signs of rejection visually as well as via H&E staining. However, even though surviving skin grafts from the experimental groups did not show signs of rejection upon visual inspection, except for slight lesions in the remaining skin grafts of mice receiving an increased dose of Phl p 5^+^ BM cells (Group C), histological analysis showed mild to moderate inflammation, advanced parakeratosis and dense dermal and epidermal infiltration of neutrophils.

## 4. Discussion

Several strategies are currently available to counter balance an existing allergic immune response in sensitized patients. These include the repeated administration of allergens in the course of specific immunotherapy and more recently the application of biologics targeting IgE antibodies or Th2 cytokines and their receptors [31,32,33].

The results of our study demonstrate that pre-existing sensitization to an allergen poses a substantial hurdle for achieving tolerization through cell therapy. Though pre-immunization in this model was limited to a single antigen, we could not achieve a favourable change in the pre-existing secondary humoral immune response using a previously developed prophylactic cell therapy protocol together with an augmented conditioning regimen. Even though transient low level chimerism was detected in most mice, the injection of Phl p 5^+^ BM cells could not suppress allergen-specific antibody responses despite the harsh conditioning regimens used. Lethal irradiation in combination with BM transplantation has shown promising results in a study performed by Colson et al. in a murine model in order to overcome a pre-existing response [23]. As the use of lethal irradiation can only be justified in life-threatening medical indications, we used only low dose, i.e., non-lethal, total body irradiation, in our protocol, which, however, was not sufficient to decrease Phl p 5-specific humoral immune responses.

The implementation of ATG in our protocol showed some positive effect in terms of Phl p 5^+^ BM cell survival after cell therapy, probably due to reduced T cell-mediated rejection of Phl p 5^+^ cells as a result of ATG treatment. Nevertheless, the chimerism that developed was low and short-lived. Even though ATG significantly decreases T cell levels, it preferentially affects naïve T cells rather than the memory T cell subset, solving only partially the problem of a pre-existing antigen-specific T cell response [34]. While skin graft survival was significantly prolonged due to transfer of increased numbers of Phl p 5^+^ BMC as well as the lack of immunization events after cell therapy, histological analysis of the surviving grafts of pre-immunized mice showed signs of infiltration of immune cells. This infiltration was not seen in skin grafts isolated from naïve mice receiving a similar treatment regimen leading to a stable and long-lived chimerism beforehand.

However, the development of Phl p 5-specific antibody responses in our model deserves some attention. In contrast to a prophylactic cell therapy approach, the intravenous transfer of Phl p 5-expressing BM cells efficiently boosted Phl p 5- but not Bet v 1-specific IgE responses, though these mice were under strong T cell-targeting immune suppression including depletion of CD3^+^ T cells by ATG and irradiation, and functional suppression by costimulation blockade. We previously demonstrated in a mouse model that allergen-specific IgE responses could be boosted by repetitive IgE epitopes without T cell help, which might also apply to the current model [35]. Secondary Phl p 5-specific IgG_1_ responses remained unaffected after cell transfer, an effect that was also observed in pollen allergic patients after seasonal allergen exposure [36]. Furthermore, following the various immunosuppressive conditioning regimens, Bet v 1-specific IgG_1_ even declined after cell transfer, which additionally indicates that the observed changes in Phl p 5-specific immune responses were caused by the administered cell therapy. 

Our experiments demonstrate the challenge of weakening or even defeating an existing immune response and emphasizes that a preventive treatment is substantially more promising and desirable given the state of knowledge and available treatment options because harsh treatment regimens can be avoided [19,20,27]. A limitation of our study was the lack of depletion of allergen-specific B cells or allergen-specific IgE and IgG antibodies which is difficult to achieve in a mouse model. Therefore, we could not fully attribute the rejection of transferred Phl p 5^+^ cells to either allergen-specific B or T cells.

## 5. Conclusions

With regard to potential translation to the clinical setting, our results indicate that cell therapy in its present form is not suitable as a therapeutic approach in the foreseeable future. In order to achieve the goal of tolerizing pre-immunized individuals, drugs or cell therapies selectively targeting B cells producing certain antibody specificities as well as antigen-specific T cells would be crucial [37]. However, the proof that robust tolerance can be achieved by transfer of either allergen-specific BM cells or B cells in naïve mice gives hope for future clinically applicable methods focusing on prophylactic treatment options for allergy [19,27].

## Figures and Tables

**Table 1:** Experimental Groups (ATG, anti-thymocyte globulin; BMC, bone marrow cells; immune, immunization).

				Immunization		Cell Transfer	Treatment				
				Pre	Post	Phl p 5 tg BMC	MR1	CTLA4Ig	Rapamycin	ATG	Irradiation
	Group	*n*=				25 × 10^6^	1 mg	0.5 mg	0.1 mg	0.45 mg	2Gy
**A**	**pre/no post imm**	9	**experimental**	**+**	**-**	day 0	**+**	**+**	**+**	**+**	**+**
**B**	**pre/post imm, treatment w/o ATG**	4		**+**	**+**	day 0	**+**	**+**	**+**	**-**	**+**
**C**	**pre/post imm, 2× BMC**	5		**+**	**+**	day 0, day5	**+**	**+**	**+**	**+**	**+**
**D**	**pre/post imm, 1× BMC**	10		**+**	**+**	day 0	**+**	**+**	**+**	**+**	**+**
**E**	**no pre/post imm**	5	**controls**	**-**	**+**	day 0	**+**	**+**	**+**	**+**	**+**
**F**	**pre/post imm, no treatment**	6		**+**	**+**	-	**-**	**-**	**-**	**-**	**-**
**G**	**pre/no post imm, no treatment**	10		**+**	**-**	-	**-**	**-**	**-**	**-**	**-**

## Data Availability

The raw data supporting the conclusions of this article will be made available by the authors on request.

## Supplementary Materials

The following supporting information can be downloaded at: https://www.mdpi.com/article/10.3390/antib13020048/s1; Figure S1 Development of Phl p 5- and Bet v 1- specific IgG1 before cell transfer; Figure S2 Changes in Bet v 1-specific IgG1 and IgE levels after Phl p 5-specific cell therapy; Figure S3 Development of a robust long-lived Phl p 5+ donor chimerism in naive mice receiving Phl p 5+ cell transfer in combination with the same treatment as the experimental groups.

## Abbreviations

ABTS	2,2′-azino-bis(3-ethylbenzothiazoline-6-sulfonic acid
ATG	anti thymocyte globulin
BM	bone marrow
BMC	bone marrow cells
ELISA	Enzyme-Linked Immunosorbent Assay
HE	hematoxilin and eosin
HRP	horseradish peroxidase
HSC	hematopoietic stem cells
IgE	immunoglobulin E
IgG1	immunoglobulin G1
i.p.	intraperitoneal
IQR	interquartile range
i.v.	intranveous
NHP	non-human primates
RBL	rat basophil leukemia
s.c.	subcutaneous
SD	standard deviation
