# Supplementary material for: Adoptive Cell Therapy in Mice Sensitized to a Grass Pollen Allergen
Source: Antibodies (Basel). 2024 Jun 18;13(2):48. doi: 10.3390/antib13020048 (PMC11200577; doi:10.3390/antib13020048)
Supplement: Supplementary file 1 [file antibodies-13-00048-s001.zip › antibodies-2963014-supplementary.pdf]

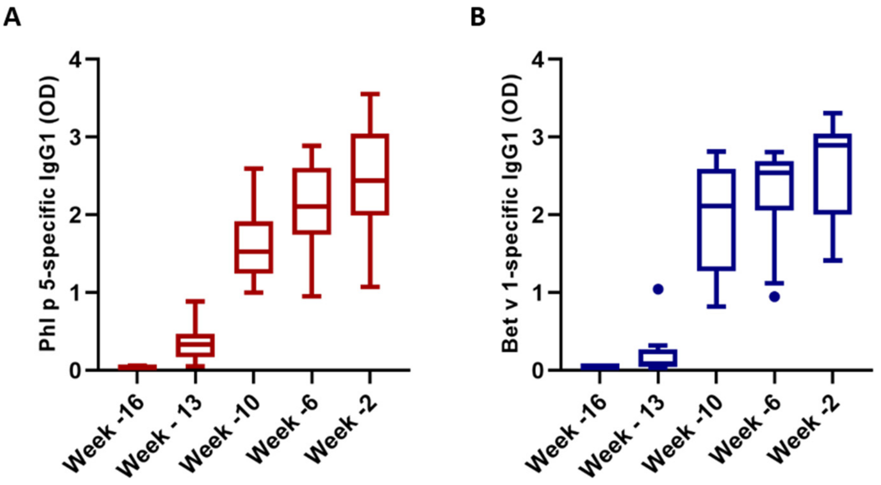

Supplementary Figure S1 Development of Phl p 5- and Bet v 1- specific IgG1 before cell transfer Pre-immunization of experimental groups was confirmed by measurement of IgG1antibodies against Phl p 5 (**A**) and Bet v 1 (**B**) as control allergen via ELISA.

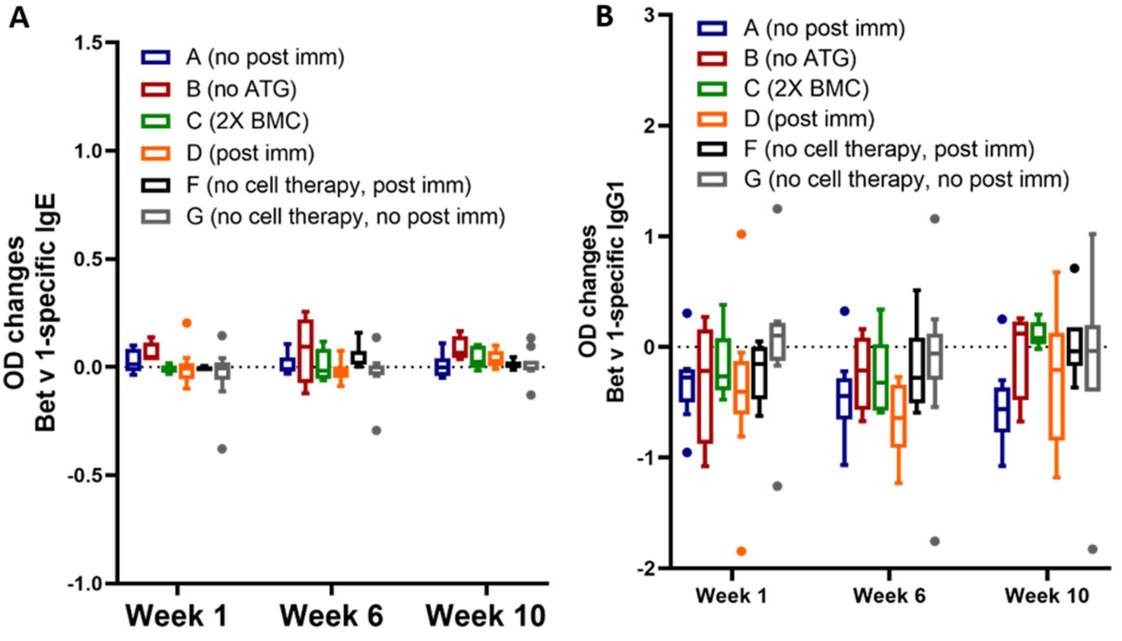

Supplementary Figure S2 Changes in Bet v 1-specific IgG1 and IgE levels after Phl p 5-specific cell therapy Bet v 1-specific IgG_1 (**A**) and IgE (**B**) levels were measured via ELISA as control to Phl p 5-specific cell treatment: Changes of antibody levels compared to levels before Phl p 5-specific cell treatment are shown. Therefore, OD values of antibody levels in serum isolated 1, 6 and 10 weeks after cell transfer were subtracted with OD values measured in serum 2 weeks before cell transfer. A n=9, B n=4, C n=5, D n=10, F n=6, G n=10

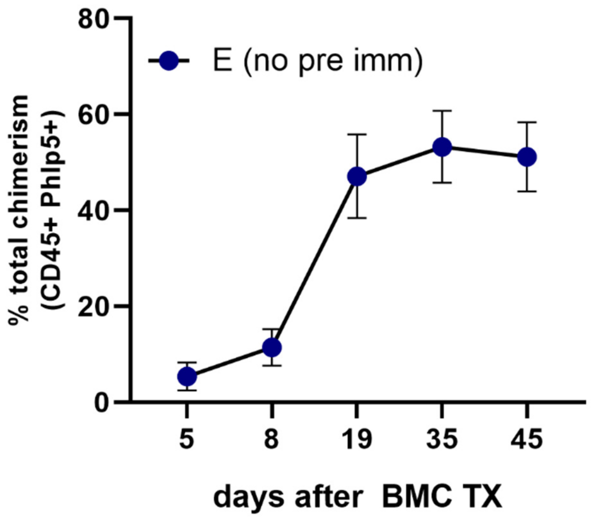

Supplementary Figure S2 Development of a robust long-lived Phl p 5$^+$ donor chimerism in naive mice receiving Phl p 5$^+$ cell transfer in combination with the same treatment as the experimental groups. Percentage of Phl p 5/GFP$^+$ cells in CD45+ cell population is shown. n=5
